# The Link between Obesity, Microbiota Dysbiosis, and Neurodegenerative Pathogenesis

**DOI:** 10.3390/diseases9030045

**Published:** 2021-06-23

**Authors:** Emanuel Vamanu, Sachchida Nand Rai

**Affiliations:** 1 Faculty of Biotechnology, the University of Agronomic Science and Veterinary Medicine, 59 Marasti blvd, 1 District, 011464 Bucharest, Romania; 2 Centre of Biotechnology, University of Allahabad, Prayagraj 211002, India; raibiochem@gmail.com

**Keywords:** gut-brain axis, neurotransmitter, dysbiosis, obesity

## Abstract

Current research in medicine in several parts of the world has attempted to establish a link between the occurrence of neurodegenerative pathologies, microbiota dysbiosis, and the incidence of obesity. The body’s response to different physicochemical factors has also been influenced by the proper assimilation of bioactive compounds contained in the food that is ingested. Oxidative stress is one of the major factors that directly affects the functioning of the human microbiota. The body’s reaction to this imbalance is crucial to the progression of inflammatory processes, which are based on molecular mechanisms. Microbial dysbiosis can result in a possibly permanent alteration in the physiological response. This review aims to highlight recent contributions made to alleviating human dysbiosis in degenerative diseases, especially for neurodegenerative pathologies based on the rising prevalence of obesity. We discuss the significance of both microbiota modulation and possible alleviations of pathologies by a modulatory function. We argue that pre- and probiotics (including phenolic compounds stimulating the favorable strain from the microbiota) are an effective alternative that can support the microbiota pattern’s modulation over time and the attenuation of indirect causes that determine dysbiosis. Molecular aspects are presented in support of the modulating role of the microbiota following the use of probiotics.

## 1. Introduction

Recovery from dysbiosis (the imbalance in gut microbial population) and the establishment of eubiosis are currently being investigated in research regarding a demonstration of a direct relationship between the nervous system and the colon’s microbial fingerprint [1]. Considered to be the second brain (enteric nervous system), the structure of enteric neurons is the key factor that controls the physiological response to the environment [2]. The immune response, an essential physiological process in maintaining homeostasis, is a consequence of the microbiota’s function [3]. Increased administration of antibiotics determines the prevalence of antibiotic resistance genes in gut bacterial strains, and sustains dysbiosis [4]. Molecular interactions between microbial strains play an essential role in the stress response, in developing pathologies, and in reducing inflammatory progression, and are a significant factor in supporting and maintaining dysbiosis [5].

Oxidative stress represents one major factor that directly affects the structure (microbial pattern) of human microbiota [6]. The perturbed pattern determines the incidence of degenerative diseases, obesity, and other physiological modifications [7].

The regenerative (modulatory) function of microbiota has been recently demonstrated in research to reduce significant pathology symptoms, mainly those involved in degenerative diseases [3]. This aspect results from microbiota-targeted therapies that modulate the microbial pattern and influence the host homeostasis [8]. Restoring the microbiota will lead to better overall functioning, which also helps maintain the functionality of other tissues or organs [3].

This evidence linking gut microbiota to degenerative pathologies is known, and new links between these are needed in the current epidemiological context. A newly identified aspect was obesity, based on its connection with the microbiota’s role. Identifying critical mediators of this process is a novelty that will open up the possibility of new therapeutic approaches by correcting dysbiosis and reducing the inflammatory response.

One example is the successful regenerative use of probiotics that improve the cognitive status (dysfunctions of memory, thinking, or language [9]). From clinical trials in patients with neurodegenerative diseases, the effect of these products has been unclear. Positive results tend to be the improvement of other physiological parameters (anxiety reduction or biochemical parameters [10]) that act indirectly on the cognitive status [11]. Probiotics, as a viable biomass, are implied in the synthesis of short-chain fatty acids (SCFAs) [12], with direct effects on the health host. SCFAs were just key mediators, which modulate the colon’s integrity. The gut-brain axis controls the body’s response to obesity and develops degenerative pathologies [13]. The prevalence of obesity has multifactorial causes. The initial stages are developed, in many cases, at the level of the nervous system by its improper function that influences gut microbiota status.

The holistic approach of restoring the microbial pattern represents a target for current research in the field and aims this review. We discuss how microbial modulation relates to the organism’s ability to respond to the effects manifested by establishing pathologies. We also discuss critical microbial species, essential biomarkers in modulating the physiological response, and alternative methods of modulating the microbial fingerprint.

We performed a literature search using Boolean operators of original articles and review articles as a research method. The literature researches in English have been conducted in Google Scholar, Scopus, or PubMed. This manuscript’s typical search terms were novel targets in obesity, a novel study in microbiota modulation, and novel clinical evaluation and molecular pathways of neurodegenerative pathologies to find the suitable papers for this manuscript (especially between 2015 and 2020). No particular limitations were taken into consideration.

## 2. Microbiota Role in the General State of Health

The most significant microbiota, in terms of physiological relevance, are predominantly located in the colon. Its role in the general state of health is essential in defining the physiological response at all levels [14], the immune system’s activity, neurotransmitter regulation, or the activation of key antioxidant enzymes [15]. These features of the microbiota’s metabolic role maintain the integrity and regeneration processes that condition the body’s recovery from different levels [16]. These regenerative aspects are closely related to inflammatory progression, as microbial profile dynamics can ensure host homeostasis. Medication is relevant in modulating regenerative processes, the bioavailability of active ingredients depending on the colonic microbiota’s action [17]. A clear picture could be represented by microbial species’ differences to observe the microbial balance in various chronic pathologies. An example of correspondence between the microbial population of microbiota, obesity, and neurodegenerative pathologies could be the variation of *Akkermansia muciniphila* population [18]:

1. In obesity, there was an increase in the *Firmicutes:Bacteroidetes* ratio (number of the cells/mL) [16,19,20] that was similar to HIV infection cases. Variations in this rate were observed in vivo and in the case of the type 1 diabetes rat model [20,21]. A strong correlation was determined in vivo between *Akkermansia* *muciniphila* [18] and an inflammatory response in relation to obesity [21,22,23];
2. In neurodegenerative diseases, variation is more complex: for Parkinson’s disease (PD), the abundance of *Lachnospiraceae* was reduced, but *Bifidobacteriaceae* and *Akkermansia* increased [24,25]. In Alzheimer’s disease (AD), increases in *Rikenellaceae* and a decrease in *Allobacillum* and *Akkermansia* were determined [26]. The relative abundances of *Akkermansia* in in vivo studies on mice exposed to chronic mild stress was 4.1% vs. 1.0% (control vs. anxiety- and depressive-like behavior; *p* < 0.001). The differences in microbial composition determined the increased abundance of pathways involved in alpha-linolenic acid metabolism, electron transfer carriers, bacterial motility proteins, Parkinson’s disease, and Prion diseases [27].

The colonic microbiota activity and the metabolic function’s modulation depend on bioactive compounds’ bioavailability [28]. Modulation by functional products influences both the microbial fingerprint, which has a xenobiotic effect, and the products of microbial metabolism. Reducing the xenobiotic impact is a complex process that affects the regenerative function [29]. Homeostasis implies a state of equilibrium between the body and the nervous system. A balance between the two systems generates a homeostatic effect. The functioning of these two systems is based on an interdependence that expresses a capacity (the physiological barrier’s function) to maintain an optimal state of health [30].

## 3. Oxidative Stress and Microbiota in Neurodegenerative Processes

Maintaining a well balanced relationship between intestinal microbiota and the nervous system is essential in promoting homeostasis [31]. It influences the general state of health, in particular, the process of neurodegeneration. This process can include any pathology that affects neuronal cells [32]. The onset of dysbiosis influences the occurrence of cognitive disorders (depression, anxiety, and associated dysfunction, such as multiple sclerosis [33]) that coincide with the onset and establishment of irritability, sensitivity, and minor neurological diseases [34]. These manifestations are sustained by inflammatory processes that characterize intestinal dysbiosis [35]. Modulation of the microbial pattern eliminates these symptoms (as multitarget strategy) [36], and classical medication of neurodegenerative pathology as AD only determines an improvement of cognitive status [37].

Inflammatory progression and aggravation reduce the natural process of neuronal protection [38], resulting in premature neuronal aging. This phenomenon frequently leads to the establishment of neurodegeneration [39]; modulation of the microbiota contributes to improving these complex processes and reducing accumulations of proinflammatory factors [3].

Nutraceuticals can be the key to this process, which controls the overall health status with long-term effects [40]. The control of these functions is also a way of reducing the natural aging process’s impact strictly related to cell oxidation and the action of oxidative stress [41]. This aspect demonstrated an imbalance between free radicals and the human body’s antioxidant mechanism [42].

Thus, antioxidants directly attenuate biological factors that can cause inflammatory progression [43]. The use of classical drugs, on the other hand, may generate toxicity and adverse reactions. Antioxidant products reduce this toxicity by the scavenging activities of free radicals. These compounds, which are often polyphenol carboxylic compounds, have other effects and, after administration, are identified as prebiotic-like products [44].

The complex relationship between biotransformation at the microbiota level and the protection against neurodegeneration results in reducing oxidative stress (an increase in antioxidant protection) and preventing the aging of nerve cells. Nutrition is key to this complicated process, but bioavailability may be limited by how the sample is processed [39]. Changing bowel motility, increased intestinal permeability, and increased drug consumption with side effects is another essential factor that correlates (or absorptive dysfunction represents) critical factors. They associate the natural aging process with a reduced immune response and an increased inflammatory progression. All of these processes are closely linked by decreasing the diversity of the microbiota pattern. The interconnections between physiological processes associated with old age progressively lead to the establishment of the neurodegeneration process [1].

The regenerative function is expressed at two levels:

1. Modulation (correction) of the disturbing pattern (dysbiosis);
2. Modulation (correction) of physiological processes resulting from restoring the balance along the gut–brain axis.

Even if the microbiota’s regenerative role is not widely accepted in clinical practice, its unbalanced metabolism favors the onset of the pathological state where oxidative stress has a more pronounced character (such as in the cardiovascular or neuronal systems) [45]. In this regard, the two functions mentioned above can be confused with each other; the two modulating features act independently. The critical point is the establishment of eubiosis, which could influence both mechanisms [46]. Metabolic correction provides a biochemical explanation for optimizing physiological functions, and the oxidative cause of stress can be eliminated [14].

Clinical relevance was determined by exo- and endogenous factors. The modulators’ chemical structure is an essential element that influences the gut-brain (biochemical signaling) balance [47]. Disruption of two-way communication is investigated in recent research, the source of neurological dysfunctions, and the cause of a decreased body immune response [48].

### 3.1. Relation with Neurodegenerative Pathologies

Microbiota activity can be controlled through the gut-brain axis using different compounds with particular functionalities based on several pathways [49] called neurotransmitters; for example, identifying the neurotransmitter pattern’s role is crucial in explaining the interaction with different exogenous factors [45,50,51].

Neuroendocrine systems also interact with the gut microbiome, controlling physiological functions in response to oxidative stress by the hypothalamic-pituitary-adrenal (HPA) axis. As different microbiota-gut-brain axis pathologies are associated with the HPA axis dysfunction [52], this interaction is significant. In PD, the triggering of limb tremors disrupts dopamine synthesis, which controls and regulates muscle movements through the gut–brain axis. The phenomenon is linked to the microbiota pattern; therefore, the microbial profile’s control determines a secondary (indirect) regulation of dopamine synthesis [53].

Recent studies have shown that microbiota plays a significant role in the pathogenesis and therapy of PD [53]. The dysbiosis of microbiota may indicate the progression of PD [54]. Several patients with PD exhibit dysbiosis and gastrointestinal problems even before the onset of motor symptoms and their diagnosis [55]. Before the clinical onset of PD symptoms, leaky gut was observed due to gastrointestinal symptoms like constipation, hypersalivation, and dysphagia [54]. The animal model and in vitro studies effectively supported these observations, highlighting α-Syn and gut dysbiosis’ role in the progression of PD [56,57].

Several gut-friendly microbes like *Lactobacilli*, *Bacteroides*, *Prevotella*, and *Bifidobacterium* were found to be decreased in patients with PD [58]. The population of pathogenic bacteria like Enterobacteria, Streptococci, Staphylococci, *Shigella*, and *H. pylori* was also increased in PD cases [59,60]. Gut microbes affect the synthesis of dopamine [61], deposition of α-synuclein enhances oxidative stress, induces local inflammation, enhances intestinal permeability, and causes constipation in PD [62,63,64]. The imbalance in the population of the bacteria would lead to dysbiosis, causing gastrointestinal tract distress like Irritable Bowel Syndrome and a dysfunctional metabolism [65,66]; therefore, gut microbiota can be explored for the early diagnosis of PD, as suggested by current studies, which are very reliable and noninvasive [67]. In PD, *Helicobacter pylori* are mentioned, with increasing levels of Enterobacteriaceae and a lower number of *Prevotellaceae* [64].

The mixture of probiotics and prebiotics might help restore the microbiota condition and improve health [68]. Some animal model studies have demonstrated that probiotics such as *Lactobacillus* could reduce LPS-induced neuroinflammation and recover memory deficits [69]. The Mediterranean diet can also sustain a healthy microbiome pattern and reduce the risk of PD by enhancing the growth of beneficial strains [55,70]; however, several side effects may be mentioned at a preprolonged administration of these functional products: intestinal bacterial overgrowth, D-lactate acidosis, brain fogginess, and horizontal gene transfer [71]. The microbiota makes a significant contribution towards the pathogenesis of PD. For example, an increased level of *Bacillus* spp. has positive effects because it increases dopamine levels [64]. The beneficial microbiome participates in a variety of functions to maintain homeostasis. The dysbiosis of microbiota may represent an early sign of PD by identifying the microbiota pattern. The gastrointestinal tract initiates a spread of α-Syn, which means that initial nonmotor symptoms like constipation might be an early sign of the disease. Several probiotics, prebiotics, and their combination might restore healthy microbiota patterns and prevent further progression of PD [53,55,72].

In AD, neurotransmitters’ activity that influences neuroinflammation directly relates to the metabolic activity [26]. Inflammation usually indicates adipocyte deposits (IL-1, TNF-α, and IL-6 that promote an inflammatory process) [26,73], and are located, especially, around the essential internal organs (e.g., liver or heart) [74]. Microbiota activity influences the formation of amyloid plaque, which progressively causes the death of neuronal cells [75]. In vivo studies have shown a decrease in certain bacteria such as *Eubacterium rectale* and *Bacteroides fragilis* and an increase in *Escherichia coli* [76].

The production of toxic compounds is a critical aspect of AD’s nervous system [37]. Once released, D-lactic acid and ammonia influence the structure and functionality of the microbial pattern. Other compounds with a negative effect are proinflammatory cytokines that cause severe inflammatory responses [77]. These aspects modify the response transmitted through the gut-brain axis, which causes memory impairment, anxiety, and other cognitive dysfunctions [78]. Increased synthesis of proinflammatory cytokines such as TNF-α, IFN-γ, IL-1β, IL-6, and IL-18 has been identified in people diagnosed with AD. Of these, two proinflammatory cytokines (TNF and IL-1β) were determined to have elevated values demonstrating the severity of AD [79]. The presence of these cytokines is correlated with the loss of the ratio between *E. coli* and *E. rectale* (the ratio is different between target groups, daily habits, etc.), which supports a typical microbial pattern in the case of degenerative diseases [80].

Amyotrophic lateral sclerosis (ALS) determines changes in the microbiota structure and dysfunction of the gut-brain axis [81]. It has been demonstrated that *Akkermansia muciniphila* reduces ALS symptoms by accumulating nicotinamides that improve motor function. An inverse effect was associated with the presence of *Ruminococcus torques* and *Parabacteroides distasonis* [82].

Based on this knowledge, we could consider that modulation of microbiota could sustain neuronal health as an alternative strategy for the population’s target group. Unbalanced metabolism favors the onset of the pathological state where oxidative stress has a more pronounced character (such as in the cardiovascular or neuronal systems). The control through the gut-brain axis involves several molecules from different pathways that do not have a well described mechanism. It is necessary to identify new biomarkers and therapeutic strategies for early diagnosis and innovative treatments, especially in preventing neurodegenerative diseases. An example of this is represented by functional products, like pro- and prebiotics, that modulate the human microbiota’s dysbiotic phases relevant to metabolic or chronic dysfunctions [83].

### 3.2. Relationship with Obesity and Type 2 Diabetes

Food addiction and drug use have a negative influence, leading to a disturbance of the neural system’s functionality. This behavior, doubled by the high degree of food processing, leads to obesity and other degenerative pathologies [84]. The initiation of dysbiosis, a result of fast-food consumption, is related to disruption in dopamine release [85,86].

Recent studies have shown a connection between obesity, type 2 diabetes, and neurodegenerative diseases. Modulation of the microbiota associated with weight loss is among the new therapeutic strategies against neurodegenerative disorders [87]. Obesity is a risk factor in developing neurodegenerative diseases (e.g., AD or PD), with common causes such as oxidative stress and mitochondrial dysfunction, both of which are supported by inflammatory progression. From a molecular perspective, early aging causes an increase in the blood-brain barrier’s permeability and intestinal permeability in the elderly, which has a proinflammatory effect [88,89].

The relationship between different bacterial strains and a dysbiotic microbiome state responds to exogenous factors and inflammatory processes generated by oxidative stress [90]. *E. coli* seems to be a key strain in this fragile balance, as evidenced by conflicting studies. A decrease in *Bacteroides* and an increase in *E. coli* have been reported in overweight women [91]. These data are correlated with recurrent infections caused by this strain in the urinary tract in people diagnosed with type 2 diabetes who are also overweight [92]. The multiplication of this strain determines a synthesis of H_2_S in the colon, which negatively influences the activity at the mitochondrial and microbiota pattern. A reduction in oxygen consumption and overexpression of proinflammatory mediator genes (e.g., IL-6) has been demonstrated [6].

Such processes induce oxidative stress, which controls several genes’ expression including those responsible for producing various cytokines [53]. The cellular level of ROS conditions the physiological response that controls inflammatory processes. For neurodegenerative diseases (e.g., AD), redox balance is a crucial factor, and *Lactobacillus* strains play an essential role in maintaining the gut–brain axis homeostasis. In the case of a high-fat diet (in vivo studies), there was an increase in proinflammatory cytokines and inflammation, which was supported by an increase in *Enterobecteriaceae* and the ratio between *Firmicutes* to *Bacteriodetes* [93]. The different physiological dysfunction (degenerative process) is closely linked to pathogenesis of inflammatory diseases (e.g., obesity or inflammatory bowel disease) [94].

The host homeostasis was influenced not only by the level of SCFA but through other cellular components. We could mention lipopolysaccharides, peptidoglycan, or genetic material. LPS are toll-like receptors (TLR) expressed by immune cells. Maintaining intestinal health and healing the possible lesions implies microbiota-mediated TLR signals. The gut microbiota mediates the inflammatory response based on the interactions with TLRs [95]. A new approach to the influence on the microbiota may be related to the amount of lipopolysaccharides produced by bacteria (determined by the perturbed intestinal permeability), given that the amount of lipopolysaccharides in the blood was correlated with diabetes, cell death, or other diseases such as sepsis, atherosclerosis, inflammatory bowel disease, nonalcoholic fatty liver disease or neurodegenerative diseases [96,97].

Other components, such as secondary bile acids produced by microbiota action, play an essential role in controlling the production of bile acids and immunity [98]. These secondary bile acids can modulate the microbiota’s composition depending on the diet [99] and lead to dysbiosis. Studies show that SCFAs and conjugated primary bile acids act differently, the first with an anti-inflammatory role, the second with the opposite role [100]. Their metabolism is also disrupted in dysbiosis, especially for patients with high *Clostridium difficile* [75], which cannot use nutrients as a carbon source [101]. Gut microbiota is involved in tryptophan metabolism, with a role in the pathogenesis of diabetes, resulting in indoles and their derivatives [102]. Some of these compounds act as aryl hydrocarbon receptor ligands, and their low levels are indicators of metabolic disorders. Simultaneously, through SCFAs and bile acids, gut microbiota also modulates the metabolism of tryptophan to serotonin; the final product of its degradation is another parameter in metabolic diseases [103]. Morever, indoles could be identified in patients with a high level of *E. coli* strains. These data could be important because the perturbed microbiota pattern can induce T2D when determining a high level of this strain [92]. This can lead to an imbalance in the gut-brain axis through a manifestation of anxiety and mood disorders [101,104].

The point of connection of neurodegenerative diseases (e.g., AD) with the intestinal microbiota is the microbial diversity changes that are often encountered with age. Research conducted in vitro supports these data, which indicates a dysbiotic condition in type 2 diabetes, which is related to obesity [92]. In ALS, the results were significant because *A. muciniphila* is considered part of a new type of functional product used to control weight and associated pathologies (reduce glucose intolerance) [105]. These data indicate another link between degenerative pathologies (e.g., ALS) with a decrease in the abundance of bacterial strains that help control obesity.

## 4. Obesity as a Factor Affecting Status of Health

Presently, there is a direct link between the modification/alteration of the microbial pattern in the colon and the progressive increase in body weight [106]. This complex process is influenced by genetic factors supported by the high caloric intake, the consumption of food additives and sweeteners, or the administration of other products (antibiotics [107]) that lead to the development of dysbiosis [108]. In recent years, obesity and colon dysbiosis have been related to degenerative diseases [5].

Less investigated causes are Coq10 (Coenzyme Q10) loss, and physical inactivity, which implies a gradual accumulation of fat. The role of Coq10 in the progression of chronic diseases, mainly cardiovascular diseases, is a certainty. Delayed administration of supplements after chronic dysfunction does not resolve the cause [109]. This is a significant source of distrust in functional supplements. The capacity to assimilate active compounds decreases with age, and the decline of this physiological process is not well known by the public [110].

Obesity is associated with the existence of a physiological marker (interleukin-6 that appears in inflammations) that leads to the early development of degenerative dysfunctions [111]. The lack of the necessary number of hours of sleep could determine the increase in fast food consumption. The decrease in the volume of consumption of natural foods (vegetables and fruits) is one of the primary causes of the development of obesity, which is associated with a reduction in the desire (gut-brain axis) to perform energy-consuming activities [112]. Some exogenous and endogenous factors (sedentary lifestyle and/or oxidative stress) also favor degenerative processes. The onset of obesity with age is a progressive process, supported by the lack of control of oxidative stress [113].

In these complex processes, the role of the microbiota becomes significant. The metabolomic pattern in specific population groups (e.g., children up to the age of 10 years) becomes essential and correlates with certain strict groups of bacteria in the microbiota (*Bacteroides* vs. *Firmicutes*) [114]. Restoring the relationship between the two groups will affect the normalizing of the metabolomic pattern [115]. A reduction in *Bacteroides* is associated with the pathological onset of obesity, and the increase in *Lactobacillus* is a biomarker of irreversible pathophysiological phenomena [116]. Thus, the increase of these strains indicates insulin resistance, a primary cause for type 2 diabetes. These data could be used as a biomarker in earlier stages. We could mention a significant number of *Escherichia coli* strains that demonstrated a high resistance at the correction of dysbiosis [117]. The intestinal microbiome has been identified as an important factor in the progression of obesity and other nutritional pathologies with an increased incidence (e.g., diabetes at younger ages) [118]. In such situations, microbiota regeneration will not compensate for the pathophysiological disturbance [119]. Although it is the triggering cause, the regenerative role cannot be manifested by introducing a chronic pathology (e.g., high blood pressure). The attempt to correct pathophysiological decline by modulation (regeneration) of the microbiota can only function within specified limits [120]. The cause for these manifestations is unknown, but it can be assumed that cellular (tissue) restoration is not directly associated with microbial restoration (microbiota) [3]. As a result of the pattern of regeneration and the metabolomic modulation, the physiological function’s correction is not associated with direct clinical manifestations that can eliminate the administration of classic medication [121].

SCFA are carboxylic acids (acetic, propionic acid, and butyric acids), the main products obtained by fermentation, and have specific physiological roles [122]. Their action is subject to the correction of metabolomic patterns, and they have, in the background, a regenerative role of physiological functions [123]. Thus, regeneration of the microbial pattern through an increased intake of plant fibers is beneficial, reducing the level of toxicity in the human colon [124]. In a recently published study [125], the consumption of a combination of fibers with probiotics caused a normalization of metabolic function (normalization of the microbial pattern) and reduced oxidative stress. These effects resulted from multiple sources of microbiota restoration, which acted at both microbial and metabolomic levels. The physiological corrections made by the SCFA action represent the determining cause of the pathophysiological recovery at the gradual decrease of body weight in the initial stages of obesity [126]. Hormonal causes (secondary causes) cause significant water accumulation in the body, resulting from changes occurring in the thyroid gland [127]. The rebalancing cannot be achieved (unpublished, independent, clinical study) by reducing the caloric intake, altering the fermentative structure as a result of increasing the quantity of fiber and improving the brain function through the action of nutraceuticals (B vitamins) [128]. Under optimal conditions, the physiological response was associated with reducing secondary inflammatory processes, increased immune function, and reducing cognitive impairment [129].

**Figure 1:**
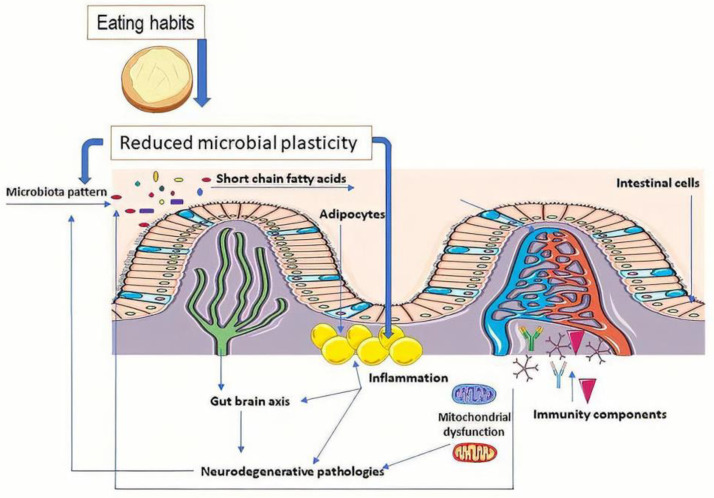
The molecular mechanism behind the role of gut microbiota in obesity and the progression of degenerative diseases. This figure was obtained in part by using images from Servier Medical Art, licensed under CC-BY 3.0, and the PowerPoint program from the Microsoft Office 2016 software package (Microsoft Corporation, Redmond, WA, USA).

Molecular research has demonstrated the role of probiotic strains (e.g., *Lactobacillus reuteri*) in the control of interleukin (IL)-10 cytokine synthesis. The process is relevant due to an anti-inflammatory effect that preserves the microbiota’s integrity, reducing dysbiosis. This strain also regulates the formation of T cells that help reduce inflammatory processes at this level [70]. The ratio between the key strains (*Bifidobacterium* spp. that inhibit T_h_17 cell and reduce metabolic inflammation) regulates the inflammatory response and controls obesity in degenerative pathologies (type 2 diabetes or neurodegenerative diseases) [67]. Altering the microbiota structure favors the expression of genes from adipocytes, a process dependent on type 2 interleukin cytokines. These lead to the activation of M2 macrophages [119]. Another in vivo study showed that the administration of *Lactobacillus curvatus* HY7601 and *Lactobacillus plantarum* KY1032 led to the regulation of proinflammatory genes (e.g., TNFα) in adipose tissue and fatty acid oxidation genes in the liver (for example, ACOX1; Figure 1) [130,131].

Controlling the amount of fat and carbohydrates in food is essential in inhibiting the tendency to increase food intake. Diet, exercise, circadian rhythms, and stressors may affect microbiota and influence the quality of sleep [132]. The interpretation of intrinsic nerve signals from the vagus nerve is lost due to sleep deprivation. This phenomenon usually occurs too late to be aware of it. When it becomes a problem, obesity is tolerated and explained by the increasing age [133]. This behavior leads to changes in the microbial pattern and the establishment of a dysbiosis that supports associated chronic diseases [134].

A significant limitation of the regenerative function comes from the activation of inflammatory processes amplified with age [135]; however, the consumption of new types of food reduces the negative role of poor nutrition in the anti-inflammatory properties of functional compounds. Substrate diversification leads to the support and promotion of a change in the microbial pattern [136]. Thus, it can be argued that diet and food quality control the microbiota structure and modulate food processing in dysbiotic states. A homeostasis can be maintained throughout the body, directly influencing the neuronal level’s functioning. An example of this would be consuming functional foods containing probiotic strains that help control obesity or reduce cognitive decline [137].

The limitation of nutrients does not maintain a positive physiological response, as plasticity occurs due to diversification. The buffering role of the microbiota pattern (which could explain the plasticity term) is managed by increasing/recognizing multiple nutrient sources, thus supporting physiological processes with the role of regulating health status [138]. Obesity is not just an outcome of inappropriate food consumption, but is the main contributing factor for modulating eating behavior and supporting dysbiosis. The reduction of *Enterobacteriaceea* in the microbiota pattern is critical for the human colon’s regenerative modulation. Recent studies have shown that this group’s role is associated with developing numerous chronic diseases and cancers, and supports cognitive decline [139]. In opposition, favorable groups are used as biomarkers for maintaining psycho-neuronal stability [140]. Although there is no definite guarantee, reducing eating habits is more important than keeping unique and constant sources of functional compounds with the microbiota pattern’s regulatory role. In all these processes, psychological stability and the desire for permanent change play an essential role in physiological regulation. The result may vary depending on the quantity and type of foods consumed, but the primary point of microbial regulation is the daily amount of food we eat [136,141].

A clear link between obesity and neural changes has been demonstrated by in vivo studies, in which the microbiota was transplanted from high-fat diet donors. The study showed that the main biomarker was influenced by microbiota, since different mental illnesses have an increased incidence of obesity [142]. Under the influence of the high-fat diet microbiota, changes in intestinal barrier function markers, increased circulating endotoxin, and an increased lymphocyte expression was identified [143]. These data were supported by improved neural inflammatory processes, which led to sustained oxidative stress. Thus, a high-fat diet leads to the growth of bone marrow-derived monocytes in the brain, demonstrating a direct link with the presence of markers of inflammation in adipose tissue (CD68 and CCL2) [144].

The human microbiota interacts with the central nervous system through the gut–brain axis [31]. The dysbiotic state disturbs this balance, and obesity is one of the leading causes of disorders that create this dysbiosis at the upper level. Through diet and the administration of functional products, rebalancing can be achieved, resulting from the administration of pro- and prebiotics [37], for example. Instead, an unhealthy diet supports peripheral inflammatory processes in Western lifestyle and increases neuroinflammation risk. Through frequent consumption of probiotics and dietary fiber, the rehabilitation of a dysbiotic pattern reduces the incidence of obesity and mental illness and leads to a regulation of the gut-brain axis [145,146].

## 5. Future Research on Microbiota Pattern

A low transit time, lack of fiber, and exercise will allow a large amount of fecal matter to remain in a latent state in the colon, favoring the retention of many harmful bacteria [147]. This phenomenon requires a physiological response from the body, i.e., increased appetite and weight gain brought about by the need to consume additional nutrients for many pathogenic bacteria which multiplied in the colon. This behavior is accompanied by an increase in ammonia. There will also be an increase in the number of strains of the genus *Lactobacillus*, leading to intolerance to insulin action and a clinical sign of type 2 diabetes [148].

Adjusting the intestinal transit and decreasing the caloric intake through diet changes, supplements to reduce the accumulated toxicity can restore eubiosis [149]. Investigating the mechanisms of protection against weight loss through metabolic interventions and caloric control leads to developing new therapeutic strategies against degenerative disorders and can increase our understanding of pathologies relevant to public health and management [87].

Chronic diseases and the strict relationship with obesity may result from microbial strains that cause inflammatory processes [131]. These processes lead to the extraction of energy from the diet, the synthesis of fats, the development of obesity, and inflammation in the fat deposit, disrupting the gut-brain axis [150]. Fostering the microbiota’s diversity increases plasticity, reduces the inflammatory process, and corrects dysbiosis [151]. This results from reducing the variety of functional foods, the accumulation of chemical products (xenobiotics), the excessive consumption of antibiotics, and the presence of oxidative stress [152]. This imbalance between free radicals and compounds with antioxidant activities represents the primary cause of cell damage [153].

The clearest implication of the relationship between diet and intestinal microbiota in pathogenesis results from in vivo studies (germ-free mouse models of human autoimmune disease), directly linking microbiota development and disease progression, has been demonstrated. The lack of microbiota determined a reduction in pathological manifestations and was considered a cause for recurrence [134]. In this situation, the microbial pattern was essential and was strictly related to eating habits [92]. Eating habits, or administered products, produce functional changes in the microbiota. The modification of key compounds (dietary fibers) stimulates certain target strains (*Lactobacillus* sp. and *Bifidobacterium* sp.). This is an effective strategy in alleviating the pathologies associated with the presence of dysbiosis [154].

The use of probiotics to increase the diversity of the colon’s microbial pattern leads to individual responses. This is an unclear point, as it is uncertain whether the new strains will cause a long-term correction of the microbial pattern [155]. Future studies will need to focus on the impact of probiotic strains or functional molecules involving a microbial or metabolomics response on the gut-brain axis. The microbiota’s role in attenuating the effects of the neurodegeneration process is another current topic that must be investigated for strains newly associated with other pathologies that depend on the onset of dysbiosis. *Akkermansia muciniphila* is one such example that can influence neuronal activity, but it is not yet clear as to how it will act in the microbiota’s environment [156].

**Figure 2:**
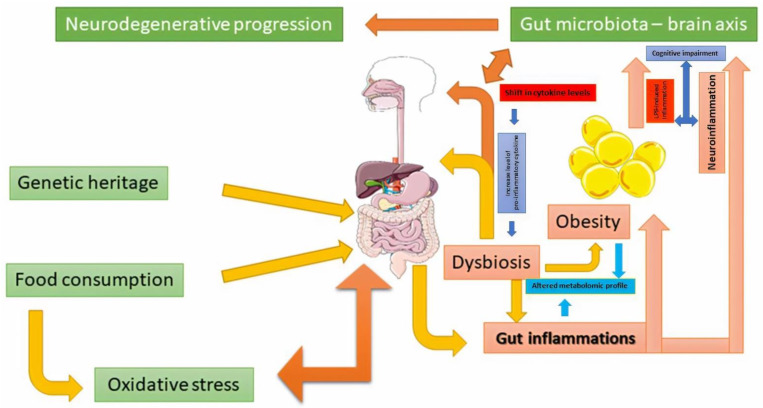
The relation between microbiota activity, obesity, and neuronal control. In this figure, food consumption and genetic heritage increased the pressure of oxidative stress at the colon level. This aspect may lead to dysbiosis and the proliferation of inflammations that induce obesity. This physiological degenerative health aspect may determine an imbalance in the gut-brain axis and develop neurodegenerative pathologies. This figure was obtained, in part, by using images from Servier Medical Art, licensed under CC-BY 3.0, and the PowerPoint program from the Microsoft Office 2016 software package (Microsoft Corporation, Redmond, WA, USA).

Neuronal diversity (different neuronal precursors) is affected by oxidative stress favored by inflammatory processes that act for prolonged periods of time [155]. Oxidative stress induces altered intracellular signaling, which leads to the degradation of physiological defense responses. The antioxidant response’s inability to modulate the proinflammatory effect has been considered an essential factor for neurodegenerative diseases [157]. In response to the increase of free radicals, the inflammatory progression is also the result of the establishment of dysbiosis, which modulates the physiological response [47] (Figure 2). Obesity (adipocyte accumulation) does not express a similar effect in risk of occurrence of all these neurodegenerative pathologies [158,159]. This could be a result of the difference between microbiota pattern and their metabolomic signature. For example, middle age obesity increases the risk of developing AD [160,161].

Demonstrating the prevalence of AD, cerebral accumulation of amyloid-β fibrils, and the relationship between dysbiosis and obesity, will be significant aspects of future research [84]. The microbiota determines the synthesis of proinflammatory cytokines that promote neurodegenerative processes [26]. Modulation of the microbiota in reducing the synthesis of the mixture of amyloids and lipopolysaccharides will represent progress in reducing fibrillogenesis and the transformation of Aβ α-synuclein fibrils into products that express cytotoxicity [162].

The theory has been demonstrated in degenerative diseases and cardiovascular pathology where the control of the microbial pattern has corrected the metabolomic response and increased antioxidant protection [158]. Thus, control of dysbiosis and risk management of oxidative stress will target the correction of physiological functions [160]. The control of diet and daily activities is specific in maintaining homeostasis, but increasing microbial diversity must be regulated by increasing microbial diversity as the key to preserving plasticity to increasingly different stressors [111,163].

The neuronal patterns play an essential role in physiological regulation, mediated by the microbiota’s functioning [39]. Microbial endocrinology (that studies the ability of microorganisms to produce and recognize neurochemicals from the microorganisms themselves or within the host [164]) aims to correct the physiological dysfunctions that occur without an exact cause [163,165]. In most studies, inflammatory progression was not correlated with weight gain, leading to a reduction in metabolites essential for maintaining homeostasis (SCFAs). This negative modeling of the microbiota led to adverse effects on the gut–brain axis and increased oxidative stress. The following steps in the field are to correlate all these data from different physiological systems. The study’s main topics will be the microbiota’s controlled modulation, correcting dysbiosis, and personalizing the medication to maintain homeostasis [156]. The onset of dysbiosis influences type 2 diabetes, and the microbiota activity is mediated by hormone-like compounds, such as dopamine and serotonin [166]. Excessive food consumption (mainly sweets, which mask hunger) creates a neural requirement to consume even more [85,91]. Neurotransmitters like dopamine control this impulse. The neuronal response modulation must also consider a change in the microbial pattern to balance along the gut-brain axis. Without restoring this balance, allopathic medication conceals many physiological imbalances [167].

Another significant aspect is the change in the response of neurotransmitters to reducing the amount of food ingested. The psycho-physiological modulation of the hunger sensation is thus controlled in the absence of dopamine synthesis, and the food control determines, over time, the regeneration of the microbial pattern. Individual variability results only from a genetic difference that controls the response along the gut–brain axis [168]. The role of neurotransmitters related to maximizing the available sources of any kind must be taken into account. Ultimately, microbiota regeneration (usually seen as a modulatory process) results from voluntary control where, indirectly, one of the control pathways at the brain level is sacrificed [169]. Neurotransmitter synthesis systems support the maximum utilization of resources, which correlates with long-term metabolic activity [170]. Changing the microbial fingerprint disrupts this process by suppressing the synthesis of one or the other of these molecules essential for human well-being [136,171].

## 6. Conclusions

We conclude that the regeneration of microbiota can start with a change in dietary style. The pattern restoration represents a stage that cannot be achieved without a difference in the mental level through limits and food restrictions. The microbiota pattern can only be adapted to a small extent by changing the metabolic response, representing a response to the human microbiome’s plasticity. Weight loss is a gradual effect that correlates with the presence of biomarkers that modulates the physiological response. The balance of the gut–brain axis has a major significance in establishing homeostasis due to neurotransmitters. The metabolic response is mediated by the presence of bioactive compounds in the diet that regulate the synthesis of critical metabolites, such as SCFAs. They are stimulated by the high consumption of polyphenols, represent a new direction after future in vitro/in vivo studies, and precisely identify the clinical relevance. In our opinion, the microbiota’s dynamic activity could be valorized in preventing the occurrence of degenerative diseases by food control and consumption of new active ingredients.

## Data Availability

Not applicable.
